# Evaluation of the Impact of Different Doses of *Curcuma longa* L. on Antioxidant Capacity: A Randomized, Double-Blind, Crossover Pilot Trial

**DOI:** 10.1155/2021/3532864

**Published:** 2021-12-14

**Authors:** Tatyanne Letícia N. Gomes, Renata Santos S. Zenha, Alisson H. Antunes, Flávia R. Faria, Kênnia R. Rezende, Evandro L. de Souza, João F. Mota

**Affiliations:** ^1^School of Nutrition, Federal University of Goiás, Goiânia, Goiás, Brazil 74.605-080; ^2^Laboratory of Biopharmacy and Pharmacokinetics, Federal University of Goiás, Goiânia, Goiás, Brazil 74.605-080; ^3^Department of Nutrition, Health Sciences Center, Federal University of Paraíba, João Pessoa, Paraíba, Brazil 58.041-900

## Abstract

Curcumin is a bioactive compound derived from *Curcuma longa* L. root, extensively studied due to its antioxidant and anti-inflammatory properties. This study evaluates the effects of different doses of powdered *C. longa* root on antioxidant capacity in healthy men. In a pilot randomized, double-blinded, crossover experiment, we acutely administered a low dose (1.5 g, LCG), moderate dose (3.0 g, MCG), and high dose (6.0 g, HCG) of *C. longa* to nine healthy men. There were no differences in plasma curcumin levels (*p* = 0.593) and antioxidant capacity (*p* = 0.473) for time × group interactions. Plasma curcumin levels increased in all groups after 20 and 90 min of *C. longa* intake (*p* < 0.05). HCG had a lower postprandial incremental area under the antioxidant capacity curve than LCG or MCG (*p* < 0.01). A low dose of *C. longa* increased the antioxidant capacity in healthy men. However, plasma curcumin levels were not dose dependently affected.

## 1. Introduction

*Curcuma longa* L. or turmeric is a perennial member of the Zingiberaceae family, widely used as a spice, food preservative, and dye [1, 2], and traditionally used as an anti-inflammatory, antioxidant, and antiseptic in Chinese and Indian medicine [3–5]. Curcumin, the major curcuminoid present in *C. longa* and responsible for the plant's yellow-orange coloration, is a low-molecular-weight polyphenol [1, 5, 6]. Curcumin pleiotropic activities have been attributed to its ability to influence multiple signaling pathways, including nuclear factor kappa B (NF-*κ*B), protein kinases, growth factor pathways, NF-E2-related factor 2 (Nrf2), and the enzymes cyclooxygenase-2 and 5-lipoxygenase [4, 5]. Curcumin can reduce oxidative stress by inhibiting NF-*κ*B-induced activation by phorbol-12-acetate-13-myristate (PMA) and hydrogen peroxide [1].

Previous experimental studies have reported the antioxidant effects of curcumin [7, 8]. However, the results of clinical investigations on the antioxidant properties of curcumin are contradicting [9–11], which could be related to the administered doses. Excessive doses of antioxidant compounds can exert contrary action increasing oxidative stress and prooxidant activity [12, 13].

This study evaluates the acute effects of different doses of *C. longa* root powder on the antioxidant capacity of healthy men. The initial hypothesis was that individuals with the highest intake of turmeric would have a greater antioxidant capacity.

## 2. Materials and Methods

### 2.1. Study Design and Participants

This is a randomized, double-blind, crossover study. An Ethics Committee of the Federal University of Goiás (Goiânia, Brazil, n. 421.008) approved the research procedure. We conducted all experiments following institutional and governmental guidelines and regulations. All participants were informed about the risks and discomforts of this study before obtaining their written informed consent. This study was registered at http://ensaiosclinicos.gov.br (n. RBR-7tj48w).

We recruited participants through social media, adverts placed on college notice boards, and flyers. Participants were healthy males from 20 to 40 years old who could attend all study assessments. For characterization, body mass index was calculated as the ratio of weight to height squared (kg/m^2^) [14]. Exclusion criteria included the use of medications or nutritional supplements, alcohol ingestion during the study, the adoption of a diet or dietary restriction, and the use of turmeric as a spice at meals in the last seven days preceding the beginning of the study.

Twelve participants were assigned to either acutely ingest different amounts of powdered *C. longa* root in capsules or a 7-day washout period between treatments [15]. The high-dose group (HCG) ingested twelve capsules containing 500 mg of *C. longa* (6 g), the moderate-dose group (MCG) ingested six capsules containing 500 mg of *C. longa* (3 g) and six containing maltodextrin, and the low-dose group (LCG) ingested three capsules containing 500 mg of *C. longa* (1.5 g) and nine capsules containing maltodextrin. Turmeric Producers Cooperative of Mara Rosa (Goiânia, Goiás, Brazil) supplied the *C. longa* root powder, and a pharmacy conducted encapsulation. The total amount of three major curcuminoids (curcumin, desmethoxycurcumin, and bisdemethoxycurcumin) found in the tested *C. longa* root powder was 4.3%, as previously reported [16].

We randomized the intervention order using random number generating software. A researcher who was not involved in the outcome evaluations performed the randomization. The treatment conditions were unknown to all participants and the research team. Codes were revealed after the completion of all analyses.

We measured plasma curcumin after administering *C. longa* capsules at three different doses (LCG, MCG, and HCG), followed by nine venous blood collection time points: before (baseline) and 20, 30, 35, 40, 45, 60, 90, and 120 min after *C. longa* capsule administration. Furthermore, we measured the antioxidant activity percentage at 0, 30, 60, 90, and 120 min after *C. longa* capsule ingestion.

### 2.2. Quantification of Plasma Curcumin Levels

Blood samples were collected using a venous catheter in heparinized tubes at nine predetermined time points. Blood was immediately centrifuged (1750 rpm, 10 min, 4°C, centrifuge Beckman, Fullerton, CA, USA) for plasma separation. Subsequently, samples were frozen and stored at −80°C in borosilicate test tubes coated in aluminum foil to avoid light exposure and degradation.

For every 2 ml of plasma, 200 *μ*l of internal standard solution (*β*-estradiol) and 1800 *μ*l of chloroform were added, vortexed (10 s), shacked (5 min), and centrifuged (5500 rpm, 10 min, 4°C). After phase separation, an aliquot (1000 *μ*l) of the organic phase was extracted and transferred to another clean borosilicate tube, followed by solvent evaporation under a nitrogen atmosphere for dryness (10 min). The dry residue was rehydrated in acetonitrile (500 *μ*l), transferred to vials, and injected into a high-performance liquid chromatography (HPLC) system. Duplicates of daily calibration curves were created for curcumin quantification and quality control samples at three concentration levels (high, medium, and low) for analytical assurance. We determined the unknown plasma curcumin concentrations using HPLC according to a validated bioanalytical method, following the European Medicines Agency guidelines [17]. All extraction procedures were conducted under low light incidence to prevent curcumin degradation.

### 2.3. Measurement of Antioxidant Capacity

The antioxidant capacity was measured using a previously described method [18], making minor modifications. The method is based on electron transfer where 2,2-diphenyl-1-picryl-hydrazine (DPPH, purple) is reduced by the action of an antioxidant to form diphenyl-picryl-hydrazine (yellow) with a consequent reduction in absorption, which can be measured by decreasing absorbance.

Initially, we prepared an 80% methanol solution to dilute 0.002 g DPPH in a 25 ml (200 *μ*mol/l) volumetric flask. After dilution, the DPPH solution was sonicated for 20 min to achieve complete solubilization. Then, 80 *μ*l of methanol representing a control sample and 80 *μ*l of methanol deproteinized plasma were dispensed into a microplate. The sample and control blank samples were repeated on the next row of the microplate. Then, 160 *μ*l of DPPH solution was dispensed in the first row, and 160 *μ*l of 80% methanol was dispensed in the second row, consecutively intercalated. After incubating samples for 50 min, we measured the absorbance at 517 nm. We calculated the percentage DPPH reduction using the following formula: percentage DPPH reduction = [1 − (the sample − A white/the control − A white)] × 100.

### 2.4. Statistical Analysis

We used Statistical Package for the Social Sciences (SPSS) statistical software v.25 (SPSS Inc., Chicago, IL, USA) for the statistical analyses. GraphPad Prism 5.0 (GraphPad Software, Inc., San Diego, CA) was used to create the graphs. Results are presented as mean ± standard deviation. We used the Kolmogorov-Smirnov test to determine the normality of the data normality. We used a two-way analysis of variance test (groups × times) with repeated measurements to analyze plasma curcumin and antioxidant capacity. When a significant interaction was detected, Bonferroni's multiple comparison method was used. We used the trapezoidal method to compare postprandial responses between conditions with the incremental area under the curve (iAUC) [19]. Effect sizes (ES) were calculated using Cohen's formula, which were then classified as small (*d* = 0.2), medium (*d* = 0.5), or large (*d* = 0.8). A *p* value of <0.05 was considered statistically significant.

## 3. Results

**Figure 1:**
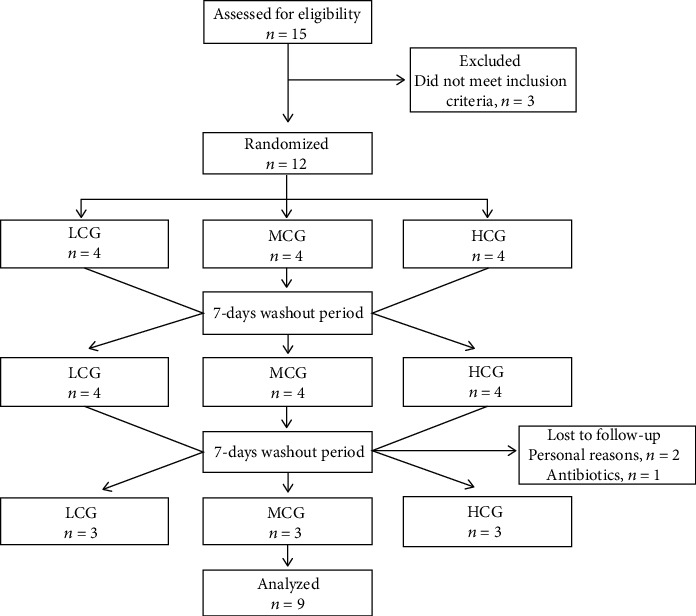
Participant flow diagram.

Twelve out of the 15 interested patients were eligible and initially enrolled in this study. Three participants withdrew for personal reasons (*n* = 2) and antibiotic use (*n* = 1) (Figure 1). The average age and body mass index of participants were 27 ± 1 y and 23.31 ± 0.75 kg/m^2^, respectively.

**Figure 2:**
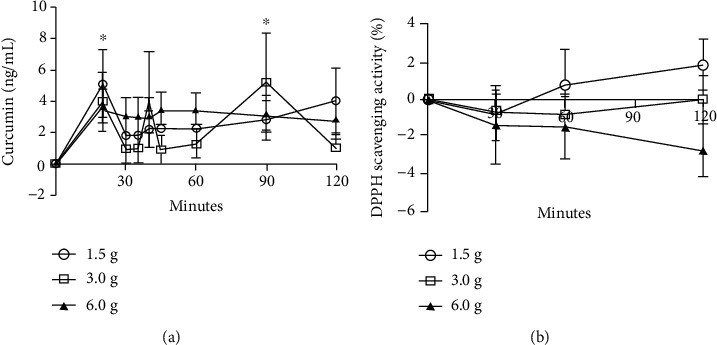
Plasma curcumin levels (a) and antioxidant capacity (b) in healthy men after acute ingestion of different doses of *Curcuma longa* L. There were no group × time interactions. ^∗^*p* < 0.05 vs. baseline in all groups.

There were no differences in plasma curcumin levels [*F* (1.66, 8.32) = 0.88, *p* = 0.637] and %DPPH [*F* (2.76, 22.09) = 1.46, *p* = 0.473] for time × group interaction. Plasma curcumin levels varied within the group, showing a significant increase after 20 and 90 min (all groups *p* < 0.05, Figure 2(a)). Ingestion of a high dose of *C. longa* did not increase %DPPH values (Figure 2(b)). However, LCG and MCG groups had better iAUC for %DPPH compared to the HCG group [*F* (2, 24) = 107.24, *p* < 0.01, ES = 0.90 and 0.82, respectively]. The maximum plasma curcumin level was 41.63 ± 87.93 ng/ml, found in the LCG group. The maximum plasma curcumin-level time was similar for the LCG and MCG groups (at 90 min), while it was found at 40 min in the HCG group (Table 1).

## 4. Discussion

In this well-controlled pilot study, we discovered that the ingestion of higher doses of *C. longa* had no significant influence on antioxidant capacity and curcumin bioavailability in healthy men. Furthermore, a low intake of *C. longa* (1.5 g) may be more effective in inducing higher antioxidant capacity. These results support the notion that the cause of oxidative stress is an imbalance between the body's prooxidant and antioxidant compounds. Thus, even nutrients with antioxidant potential could exert contrary action in the body when administered in excess [13]. Furthermore, curcumin is a nutritional hormone that follows hormonal pathways because the response to hormonal dose is biphasic and characterized by a dose-response relationship divided into two dose zones: low-dose area with stimulatory responses and high-dose area with inhibitory and adverse reactions [20].

In this study, plasma curcumin levels were undetectable at some collection points, exhibiting fluctuations throughout the study. These fluctuations could be attributed to curcumin metabolism, as the absorption of this compound is followed by conjugation in the liver (glucuronidation and sulfation) and excretion through the gallbladder. Curcumin deconjugation occurs in the intestine due to the action of intestinal bacteria and is reabsorbed [21]. Curcumin is also rapidly absorbed, metabolized, and distributed to tissues within the first few minutes [22]. Therefore, low plasma levels observed in studies using oral curcumin may be not only due to its malabsorption and rapid metabolism but also due to rapid tissue distribution [23].

Garcea et al. [24] discovered trace amounts of curcumin (<1 ng/ml) in patients with colorectal cancer after ingestion of 3.6 g of curcumin, which is consistent with the results of this study. Similarly, another study reported that turmeric extract supplementation (440–2200 mg/d; 36–180 mg of curcumin) in 15 patients with advanced colorectal cancer for 29 days did not increase curcumin levels or the metabolites in the blood or urine [25]. However, consumption of >8 g of curcumin per day did not increase plasma curcumin levels in proportion to the administered doses in healthy volunteers [26, 27]. Thus, plasma curcumin levels did not increase proportionally to the administered dose, probably due to absorption system saturation.

In this study, the maximum plasma curcumin-level time was 90 min, which was longer than that (30–45 min) found by Pawar et al. [22]. However, these authors used curcumin in conjunction with lipid formulation. Curcumin is a highly lipophilic compound that could be absorbed faster when administered with liposomes, micelles, or phospholipid complexes [28, 29]. Curcumin absorption may reduce even further when fasting because bile synthesis required for solubilization of hydrophobic compounds is reduced [28]. In our study, capsules were ingested while fasting, which may have reduced curcuminoid absorption.

*C. longa* has been reported as a potent antioxidant and anti-inflammatory herb [30]. However, there are still disagreements among studies regarding these effects, which may be related to curcumin dose in tested supplements. It has been demonstrated that a high concentration of antioxidants can induce prooxidant activity [13]. Supporting this finding, an early experimental study showed that curcumin concentration dependently exhibits anti- and prooxidant effects [12]. In a previous study [31], a low dose of a curcumin-lipid preparation (80 mg/day) induced various potentially health-promoting effects in healthy middle-aged individuals, including free radical scavenging capacity, increased plasma catalase activity and plasma myeloperoxidase levels, and lower levels of C-reactive protein and plasma nitric oxide [31]. Curcumin administration as a supplement has been considered safe, with clinical trials in humans revealing no dose-limiting toxicity when administered up to 12 g/day [26].

This study had some limitations, such as a small number of participants despite being a pilot study. Furthermore, we evaluated the effects on healthy men without metabolic stress, being possible that individuals with oxidative stress could need higher doses of an antioxidant to reach positive effects on antioxidant capacity.

## 5. Conclusions

In conclusion, a low dose of *C. longa* enhanced the antioxidant capacity in healthy men; however, plasma curcumin levels were not dose-dependently affected. High doses of *C. longa* might negatively impact the antioxidant capacity in healthy men. Further studies with a larger sample size and longer duration are required to corroborate these results.

## Figures and Tables

**Table 1:** Pharmacokinetic parameters for plasma curcumin levels in healthy men induced by acute ingestion of different doses of *Curcuma longa* L.

	LCG (1.5 g)	MCG (3.0 g)	HCG (6.0 g)
*C*_max_ (ng/ml)	41.63 ± 87.93	41.20 ± 12.35	2.84 ± 8.53
*T*_max_	90 min	90 min	40 min

Results expressed as mean ± standard deviation. *C*_max_: maximum concentration; *T*_max_: time of maximum concentration; LCG: low dose of *C. longa* L.; MCG: moderate dose of *C. longa* L.; HCG: high dose of *C. longa* L.

## Data Availability

Data is available on request.
